# Repetitive spreading depolarization induces gene expression changes related to synaptic plasticity and neuroprotective pathways

**DOI:** 10.3389/fncel.2023.1292661

**Published:** 2023-12-14

**Authors:** Michela Dell’Orco, Jordan E. Weisend, Nora I. Perrone-Bizzozero, Andrew P. Carlson, Russell A. Morton, David N. Linsenbardt, C. William Shuttleworth

**Affiliations:** ^1^Department of Neurosciences, The University of New Mexico School of Medicine, Albuquerque, NM, United States; ^2^Department of Neurosurgery, The University of New Mexico School of Medicine, Albuquerque, NM, United States

**Keywords:** spreading depression, preconditioning, gene expression, pathway analysis, brain injury, stroke

## Abstract

Spreading depolarization (SD) is a slowly propagating wave of profound depolarization that sweeps through cortical tissue. While much emphasis has been placed on the damaging consequences of SD, there is uncertainty surrounding the potential activation of beneficial pathways such as cell survival and plasticity. The present study used unbiased assessments of gene expression to evaluate that compensatory and repair mechanisms could be recruited following SD, regardless of the induction method, which prior to this work had not been assessed. We also tested assumptions of appropriate controls and the spatial extent of expression changes that are important for *in vivo* SD models. SD clusters were induced with either KCl focal application or optogenetic stimulation in healthy mice. Cortical RNA was extracted and sequenced to identify differentially expressed genes (DEGs). SDs using both induction methods significantly upregulated 16 genes (vs. sham animals) that included the cell proliferation-related genes FOS, JUN, and DUSP6, the plasticity-related genes ARC and HOMER1, and the inflammation-related genes PTGS2, EGR2, and NR4A1. The contralateral hemisphere is commonly used as control tissue for DEG studies, but its activity could be modified by near-global disruption of activity in the adjacent brain. We found 21 upregulated genes when comparing SD-involved cortex vs. tissue from the contralateral hemisphere of the same animals. Interestingly, there was almost complete overlap (21/16) with the DEGs identified using sham controls. Neuronal activity also differs in SD initiation zones, where sustained global depolarization is required to initiate propagating events. We found that gene expression varied as a function of the distance from the SD initiation site, with greater expression differences observed in regions further away. Functional and pathway enrichment analyses identified axonogenesis, branching, neuritogenesis, and dendritic growth as significantly enriched in overlapping DEGs. Increased expression of SD-induced genes was also associated with predicted inhibition of pathways associated with cell death, and apoptosis. These results identify novel biological pathways that could be involved in plasticity and/or circuit modification in brain tissue impacted by SD. These results also identify novel functional targets that could be tested to determine potential roles in the recovery and survival of peri-infarct tissues.

## 1 Introduction

Spreading Depolarization (SD) is a slowly propagating wave of extended depolarization that is followed by transient suppression of neural activity, first described by Leao (1944). SD is implicated as a mechanism of migraine with aura (Charles and Brennan, 2009; Pietrobon and Moskowitz, 2014) and otherwise healthy brain is able to fully recover from SD (Nedergaard and Hansen, 1988). However, the long duration of depolarization (up to a minute or more) can lead to severe energy depletion (Lindquist and Shuttleworth, 2014; Dreier et al., 2017), particularly when coupled with neurovascular disruptions in ischemic brain (Dreier, 2011; Ayata and Lauritzen, 2015; Hartings et al., 2017b). In addition, transient extracellular glutamate surges caused by SD can be sufficient to cause neuronal injury in metabolically compromised brain tissue (Aiba et al., 2012; Hinzman et al., 2015). Recent work has identified SDs as contributors to tissue loss following stroke, trauma, and other acute brain injuries (Lauritzen et al., 2011; Dreier et al., 2017) and initial efforts have begun targeting SD in efforts to improve outcomes in brain injury (Carlson et al., 2018; Helbok et al., 2020).

In contrast to the large body of literature characterizing electrophysiological and vascular responses to SD, only a relatively small number of studies have examined SD-induced gene expression changes and their relationship to outcomes in different experimental and clinical conditions (Urbach et al., 2006; Sintas et al., 2017). Initial studies identified increases in brain derived neurotrophic factor (BDNF), c-Fos and heat shock protein 70 (HSP70) (Kokaia et al., 1993; Matsushima et al., 1996; Karikó et al., 1998; Dietrich et al., 2000; Rangel et al., 2001). Subsequent studies showed increased expression of pro-inflammatory and apoptotic genes, including tumor necrosis factor (TNF), C-C motif chemokine ligand 2 (CCL2), interleukin-1 (IL-1) and -6, and cyclooxygenase-2 (COX-2), and B-cell lymphoma 2 (BCL-2) (Jander et al., 2001; Kaido et al., 2012; Eising et al., 2017; Takizawa et al., 2020). While most of these studies focused on a predetermined set of genes, a microarray study applied gene ontology (GO) and pathway analysis (PA) to examine the effect of SDs in rats (Sintas et al., 2017). This study identified upregulation of genes in pathways linked to oxidative stress and cellular injury, but less is known of other cellular adaptations that may occur. A lack of comprehensive transcriptomic analysis has limited the understanding of potentially beneficial pathways that could be activated following SD.

In the current study, we provide the first unbiased RNAseq analysis following SD. To increase generalizability, we combined data from the two most common methods of SD initiation (focal depolarization with either topical KCl administration or optogenetic stimulation) and generated clusters of SDs that are analogous to patterns of SDs that have been recorded from injured human brain. We also examined two key issues specific this type of study in SD models; (1) appropriate control tissues; and (2) relative contribution of initiation and propagation sites to gene expression changes. Finally, we applied pathway analysis approaches to uncover candidate networks and novel genes altered by SD. The results identify the upregulation of multiple molecular pathways in the propagation zones of SD that could regulate potentially beneficial effects, such as neuritogenesis and synaptic plasticity.

## 2 Materials and methods

### 2.1 Animal model

All experimental procedures were performed in accordance with the National Institutes of Health Guide for Care and Use of Laboratory Animals and were approved by the University of New Mexico Health Sciences Center Institutional Animal Care and Use Committee (IACUC). All studies were conducted in in healthy 7-8-week-old female mice, anesthetized with 1.5 mg/g (i.p.) urethane (Sigma-Aldrich) full dose injection followed by 0.75 mg/g (i.p.) half dose after 20 min. Animals were then placed in a stereotaxic frame and remained under anesthesia for the entirety of the experiment; body temperature was maintained 37 ± 0.5°C. A skin flap was created to expose the skull, and mineral oil placed to facilitate 2-D intrinsic optical imaging (IOS). The exposed cortex was illuminated with white light collected through a CCD camera; the changes in absorption and reflection of white light due to SD propagation allow SDs detection with non-fluorescence imaging (Hillman, 2007; Pinkowski et al., 2023). We used a Nikon SLR camera NIKKOR 50 mm f/1.8–16 lens (f-stop for imaging = 5.6) and a 12 mm extension tube was attached to a Mightex USB CCD (CXE-B013-U). IOS signals were then monitored in real time and recorded for 2.5 min. Light intensities were converted to a 16-bit grayscale image by the CCD camera, and SDs propagation images were generated after normalization and ΔF/F_0_ analysis (Image J 1.53e)^1^ to ensure SD generation (Supplementary Figure 1 and Supplementary Videos 1, 2). In some animals, a burr hole was prepared to allow for subsequent KCl application to induce SD (see below).

Spreading depolarizations were then induced repetitively (4 SDs in 2 h at 30 min intervals) SDs were induced in 6 mice with either focal application of KCl (1M) through a burr hole in C57Bl6J mice (*n* = 3) or with optogenetic stimulation (3 mW for 20 s) in Thy1-ChR2-YFP positive mice (*n* = 3). Thy1-ChR2-YFP breeders were initially purchased from The Jackson Laboratory (B6.Cg-Tg(Thy1-COP4/EYFP)18Gfng/J, Strain #:007612, RRID:IMSR_JAX:007612) and crossed to maintain the line, as previously described (Arenkiel et al., 2007). With either stimulus, evoked SDs propagated throughout the neocortex ipsilateral to the stimulus, without crossing to the contralateral hemisphere (Supplementary Figure 1 and Supplementary Videos 1, 2). A matched number of sham controls was used with focal NaCl administration through a burr hole, in C57Bl/6 mice (*n* = 3), or 0.4 mW for 20 s in Thy1-ChR2-YFP positive mice (*n* = 3). Thirty min after the last SD, the anesthetized animal was decapitated and the brain extracted, washed with ice cold PBS and kept on ice.

### 2.2 RNA extraction

The contralateral and ipsilateral cortices were rapidly dissected from underlying striatum and the hippocampus and total RNA was extracted using TRIzol (Invitrogen, Thermo Fisher, Waltham, MA, USA). Extractions were either from intact ipsilateral or contralateral cortices (Figures 2–6) or from regions ∼18 mm^2^ rapidly micro-dissected on ice (Figure 6). Where indicated, microdissections were made from 3 different regions of the ipsilateral hemisphere: (1) SD initiation site; (2) intermediate site >3 mm from initiation, and (3) remote site >5 mm from initiation (Supplementary Figure 3). All collected tissues were then flash frozen and kept at –80°C until RNA extraction. RNA was quantified using Qubit (Bio-Rad) and its quality was determined using a NanoDrop 1000 (Thermo Fisher), using absorbance at 260, 280 and 230 nm. Aliquots of 2 μg RNA were sent to Arraystar, Inc. for Illumina paired-end RNAseq as described below.

### 2.3 RNA sequencing

RNAseq analysis was performed by Arraystar Inc. (Rockville, MD, USA). Total RNA (2 μg) was used to prepare the sequencing library in the following steps: total RNA was enriched by oligo (dT) magnetic beads (rRNA removed); RNAseq library was prepared using KAPA Stranded RNASeq Library Prep Kit (Illumina), which incorporates dUTP into the second cDNA strand and renders the RNAseq library strand-specific. The completed libraries were qualified with an Agilent 2100 Bioanalyzer and quantified by using the absolute quantification qPCR method. To sequence the libraries on an Illumina NovaSeq 6000 instrument, the barcoded libraries were mixed, denatured to single stranded DNA in NaOH, captured on Illumina flow cell, amplified *in situ*, and subsequently sequenced for 150 cycles for both ends on an Illumina NovaSeq 6000 instrument. Sequence quality was examined using the FastQC software. The trimmed reads (trimmed 5′, 3′-adaptor bases using cutadapt) were aligned to a reference genome (Mouse genome GRCm38) using Hisat2 software.

### 2.4 Differential expression analysis

The expression levels of known genes and transcripts were calculated using the StringTie and Ballgown software packages (Pertea et al., 2016) and expressed as fragments per kilobase of transcript per million mapped reads (FPKM). The number of identified genes and transcripts per group was calculated based on the mean of FPKM in group >0.5. Prior to performing DESeq2 analysis, genes were filtered to eliminate genes with less than 0.5. counts per million. Differentially expressed gene (DEG) and transcript analyses were performed using unpaired *t*-tests followed by Welch’s correction; a fold change (FC) threshold of 1.25 and *p*-value < 0.05 were used together to classify genes and transcripts as differentially expressed. We subsequently performed DESeq2 analysis (Ge et al., 2018) with a false discovery rate (FDR) adjusted *p*-value cutoff of < 0.01 and FC > 1.5. We then performed a *post hoc* power analysis: we estimated that with a n of 6 and alpha error of 0.05, the effect size is equivalent to a Cohen’s *d* of 5.47 (or a rho of 0.939) (Rothstein et al., 1992).

### 2.5 qPCR validation

To validate gene expression from our RNAseq data set, separate aliquots containing 1 μg of total RNA were reverse transcribed using SuperScript II reverse transcriptase (Life Technologies) following the manufacturer’s protocol and qPCR carried out with a CFX96 Touch Real-Time PCR Detection System using SYBR Green mix (Life Technologies). Primer sequences for qRT-PCR for mouse mRNAs were obtained from PrimerBank.^2^ The GAPDH gene (FW TGTGATGGGTGTGAACCACGAGAA, RV GAGCCCTTCCACAATGCCAAAGTT) was used to normalize values. As shown in Supplementary Figure 3, GAPDH expression itself was unchanged by SD with either KCl or optogenetic induction of SD. The relative expression of target genes was determined using the comparative 2-ΔCt method (Livak and Schmittgen, 2001).

Values were expressed as means ± SEM. Statistical analysis was performed using GraphPad Prism version 9 (La Jolla, CA, USA). The data were analyzed by unpaired *t*-test followed by Welch’s correction or analysis of variance (ANOVA) followed by Dunnett’s Multiple Comparison tests. Differences were considered statistically significant when *p*-values were < 0.05 (*p < 0.05, **p < 0.01, ***p < 0.001). We performed a *post hoc* power analysis: we estimated that with a n of 12, and alpha error of 0.05, the effect size is equivalent to Cohen’s *d* of 2.37, or a rho value of 0.99 (Rothstein et al., 1992).

### 2.6 Ingenuity pathway analysis

Predicted molecular functions and biological pathways enriched in genes differentially expressed after SD were identified using Ingenuity Pathway software [IPA, Content version: 62089861 (Release Date: 2021-02-17), Qiagen, (Krämer et al., 2014)].^3^ The analysis was set to filter nervous system expressed genes only; reference set: Ingenuity Knowledge Base (Genes Only); and including direct and indirect relationships.

## 3 Results

### 3.1 Identification of gene expression induced by repetitive spreading depolarizations

Healthy female mice were subjected to multiple SDs (4 over a period of 2 h) which were generated to match clusters of SDs in clinical settings (Dreier et al., 2017). This procedure has previously been shown to induce robust transcriptional changes (Urbach et al., 2006; Sintas et al., 2017; Takizawa et al., 2020). Thirty minutes after the last SD or sham stimulus, the neocortex was removed, and RNA extracted from tissue subjected to SD or shams for subsequent RNA sequencing (see section “2 Materials and methods and Supplementary Figure 1).

**FIGURE 1:**
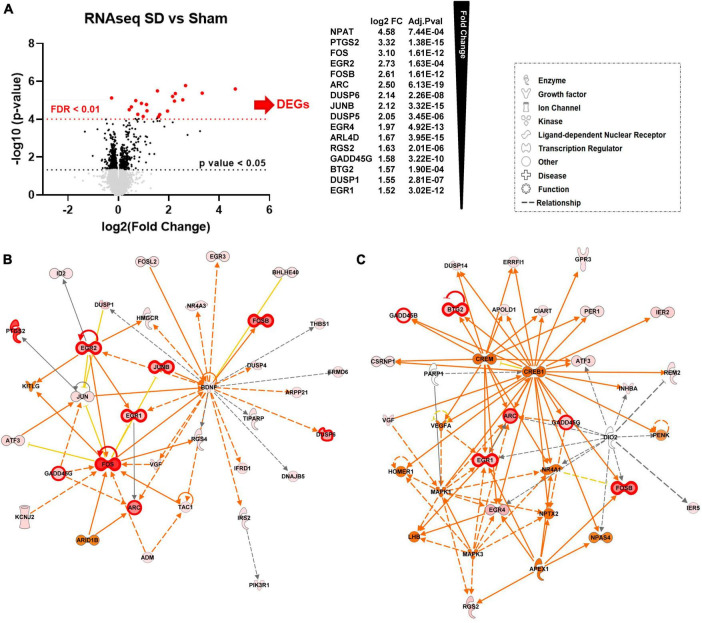
Repetitive SD increases levels of genes related to nervous system development and function **(A)** Differential expression (DE) analysis. We performed unpaired *t*-test followed by Welch’s correction; significant genes (FC > 1.25, *p*-value < 0.05, *n* = 6) are represented in black. Consequently, we performed DESeq2 analysis (Ge et al., 2018); genes that were also significant after false discovery rate correction (FDR adjusted *p*-value < 0.01, *n* = 6) are represented in red. **(B,C)** Top molecular networks (score 63 and 32) generated by IPA analysis of DEGs upregulated in SD animals vs. sham. The intensity of the color red for each molecule is proportional to the FC of the gene. Genes with FDR < 0.01 are highlighted with a thicker red outline. Full arrows indicate a direct relationship between molecules, while dotted arrows indicate an indirect relationship between genes. Orange arrows and molecules indicate the predicted activation of a gene, while yellow indicates predicted inhibition.

Both focal KCl and optogenetic stimulation are utilized in studies of SD in brain injury, and thus to increase generalizability of this initial study of SD regulation of target genes, data from these two SD-stimulation methods were combined for differential expression (DE) analysis. For the combined DE analysis, we performed unpaired *t*-tests followed by Welch’s correction on the mapped 12,944 genes. We identified 102 significantly differentially expressed genes (DEGs) (Figure 1A, black, FC > 1.25, *p*-value < 0.05, *n* = 6, Supplementary Table 1). We subsequently performed DESeq2 analysis (Ge et al., 2018) and identified 16 genes that were also significant after false discovery rate correction (FDR adjusted *p*-value < 0.01, FC > 1.5) when comparing the SD group with the sham tissues (Figure 1A, red, FDR < 0.01, FC > 1.5, *n* = 6, Supplementary Table 1).

Consistent with previous studies (Kokaia et al., 1993; Dietrich et al., 2000; Rangel et al., 2001; Kaido et al., 2012), we found significant changes in immediate early genes (IEG) ARC, FOS, and JUN proto-oncogenes, and early growth response (EGR) genes. Other DEGs with significantly increased levels included adrenoceptor beta 1 (ADRB1), dual specificity phosphatase (DUSP) gene family, the nuclear receptor subfamily 4 group A member 1 (NR4A1), and prostaglandin-endoperoxide synthase 2 (PTGS2).

### 3.2 Molecular networks regulated by repetitive SDs

We next used Ingenuity Pathway Analysis (IPA) to identify molecular networks and biological pathways regulated by DEGs. This information was then used to obtain a prediction of physiological and pathological brain processes altered by SD. Enriched pathway analysis on FDR significant genes showed that genes upregulated by SD are involved in nervous system development and function, tissue morphology and development. We also found significant enrichment in genes involved in cell death and survival and cell morphology (Supplementary Table 2).

To achieve a more extensive enrichment analysis we then expanded IPA pathway analysis to genes with *p*-value < 0.05 and FC > 1.25. Figures 1B, C show the top two scored networks (score 63 and 35) generated by IPA with the combined DEGs upregulated in the SD group as compared to sham animals. The intensity of the color red for each molecule is proportional to the fold change (FC) of the labeled gene. Genes with FDR < 0.01 are also highlighted with thicker red outlines. Full arrows indicate a direct relationship between molecules, while dotted arrows indicate a predicted indirect relationship. Orange arrows and molecules indicate the predicted activation of a gene, while yellow indicates predicted inhibition. These networks implicated SD target genes associated with cell proliferation and synaptic activity, including cAMP responsive element binding protein 1 (CREB1), mitogen-activated protein kinase 1 and 3 (MAPK1 and MAPK3), and HOMER1 (Figure 1C).

Predictions of SD-induced changes associated with known disease states and functions are shown in Table 1 and they include: neurological diseases, psychological disorders, nervous system development and function, tissue morphology, and tissue development. The complete list of diseases and functions and the genes associated with each pathway are reported in Supplementary Table 2. Figure 1D also shows the number of genes associated with each pathway and associated *p*-value range. They are shown together so that different strengths of the network elements can be compared. A complete list of networks and associated DEGs is reported in Supplementary Table 3.

**TABLE 1:** Predictions of SD-induced changes in associated nervous system diseases and functions, in SD vs. sham animals.

Disease and disorders	*p*-value range	# Molecules
Neurological diseases	3.69E-02–7.77E-09	42
Organismal injury and abnormalities	3.69E-02–6.10E-09	43
Psychological disorders	2.65E-02–6.10E-09	29
Hereditary disorders	2.65E-02–6.10E-09	18
Skeletal and muscular disorders	2.65E-02–2.70E-08	17
**Physiological system development and functions**	***p*-value range**	**# Molecules**
Nervous system development and function	3.69E-02–7.62E-07	51
Tissue morphology	3.69E-02–4.54E-08	28
Tissue development	3.46E-02–7.62E-07	40
Organismal development	3.46E-02–4.43E-06	37
Behavior	3.17E-02–1.11E-05	12

**FIGURE 2:**
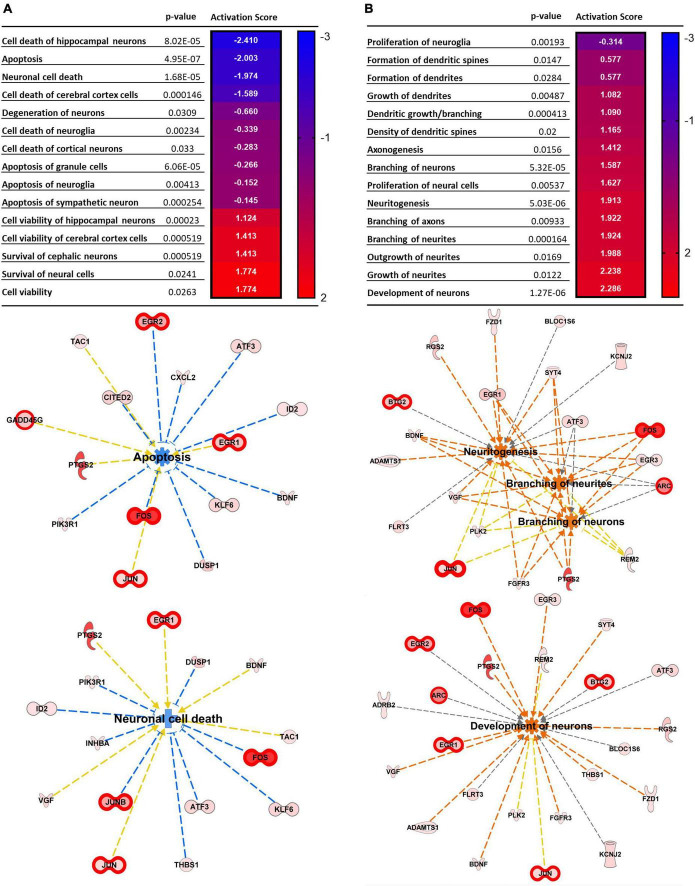
Top cellular pathways activated by SD. **(A)** Neural death and survival pathways. **(B)** Cellular proliferation and growth. Top cellular functions generated by IPA analysis of DEGs upregulated in SD animals vs. sham. The heatmaps show the *p*-value and the activation score for each pathway. **(A’,B’)** Graphical representations of the networks and the genes involved in **(A,B)**. The intensity of the color red for each molecule is proportional to the FC of the gene. Orange arrows indicate a positive control of the gene on the related pathway, and the pathway’s name highlighted in orange indicates its activation. Genes with FDR < 0.01 are also highlighted with thicker red outlines. Apoptosis z score –1.197, neuronal cell death z score –0.903, neuritogenesis z score + 2.048, branching of neurites z score + 1.924, branching of axons z score + 1.922, development of neurons z score + 2.224.

Figure 2 illustrates pathways related to cellular functions induced by repetitive spreading depolarization. The top common pathways identified include: cellular assembly and organization, cellular function and maintenance, and cellular development and proliferation (Supplementary Table 4). The heatmaps in Figures 2A, B show the *p*-value and the activation z score for each pathway, and a graphical representation of the cellular functions and genes involved, respectively. As shown in Figure 2, each gene expression FC is proportional to their color intensity; orange arrows and pathways indicate a predicted activation, whereas blue indicates increased levels of genes which lead to inhibition of the pathway; genes with FDR < 0.01 are also highlighted with thicker red outlines. IPA analysis predicted an activation of axonogenesis, axon branching, dendritic growth and branching and growth of neurites, based on increased levels of expression of genes encoding VGF, FOS, PTGS2, JUN, BDNF, gamma-aminobutyric acid type A receptor subunit beta3 (GABRB3), activating transcription factor 3 (ATF3), and cyclin dependent kinase like 3 (CDKL3). We also found predicted activation of pathways regulating neuronal development, long-term potentiation, and neurotransmission due to increased expression of FOS, PTGS2, EGR2, BDNF, JUN, KCNJ2, ARC, and HOMER1.

Interestingly, we also found predicted inhibition of neuronal cell death (z score −0.903), apoptosis (z score −1.197) (Figure 2A), together with increased development of neurons (z score + 2.224) survival of cerebral cortex cells (z score + 1.774), and cell viability (z score + 1.413) (Figure 2B), due to increased expression of genes such as FOS, BDNF, ATF3, JUNB, DUSP1, neuronal PAS domain protein 4 (NPAS4), nuclear factor interleukin 3 regulated (NFIL3), and SERPINE1.

### 3.3 Comparison between contralateral and ipsilateral hemispheres

Many studies utilize the contralateral neocortex as a convenient comparison tissue for DEG studies (Shen and Gundlach, 1998; Volobueva et al., 2023), as SD does not directly propagate across the corpus callosum. However, even if SD does not directly propagate through the contralateral cortex, the profound and widespread disruption of neuronal network activity produced by SD might be expected to have some impact on neuronal activity and gene expression in the contralateral hemisphere. The assumption that contralateral tissue is equivalent to tissue from sham animals for DE analysis has not previously been tested.

We first performed DE analysis between these 2 tissues (i.e., contralateral hemispheres in animals exposed to SD in the ipsilateral hemisphere vs. sham tissues). As shown in Supplementary Figure 2 only 9 genes were significantly differently expressed after unpaired *t*-test followed by Welch’s correction, but none of were still significant after FDR correction, confirming a high degree of similarity between gene expression in these tissues.

**FIGURE 3:**
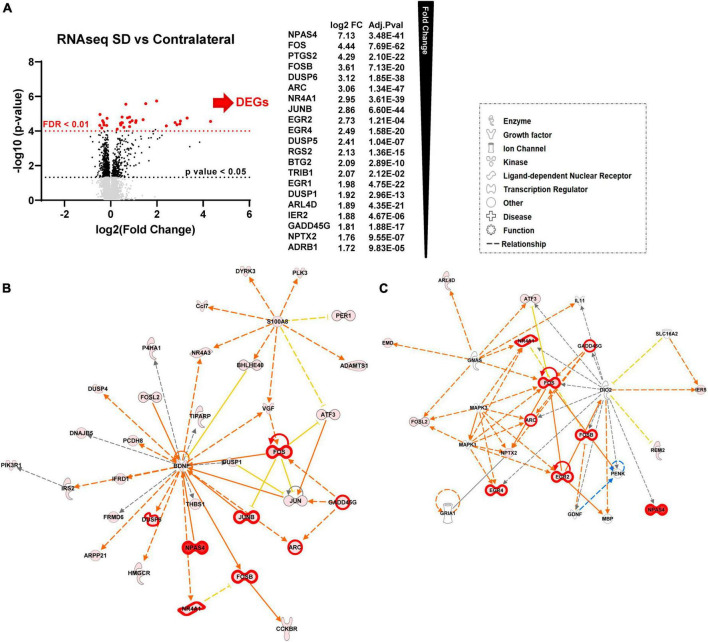
Sham animals and contralateral cortex are equivalent controls for unilateral SDs. (A) Differential expression (DE) analysis. We performed unpaired *t*-test followed by Welch’s correction; significant genes (FC > 1.25, *p*-value < 0.05, *n* = 6) are represented in black. Subsequently, we performed DESeq2 analysis (Ge et al., 2018); genes that were also significant after false discovery rate correction (FDR adjusted *p*-value < 0.01, *n* = 6) are represented in red. (B) Top molecular networks (score 65 and 34) generated by IPA analysis of DEGs upregulated in SD animals vs. contralateral. The intensity of the color red for each molecule is proportional to the FC of the gene. Full arrows indicate a direct relationship between molecules, while dotted arrows indicate an indirect relationship between genes. Orange arrows indicate the predicted activation of a gene, while yellow indicates predicted inhibition. Genes with FDR < 0.01 are also highlighted with thicker red outlines. (C) Top diseases and functions determined from IPA analysis.

**FIGURE 4:**
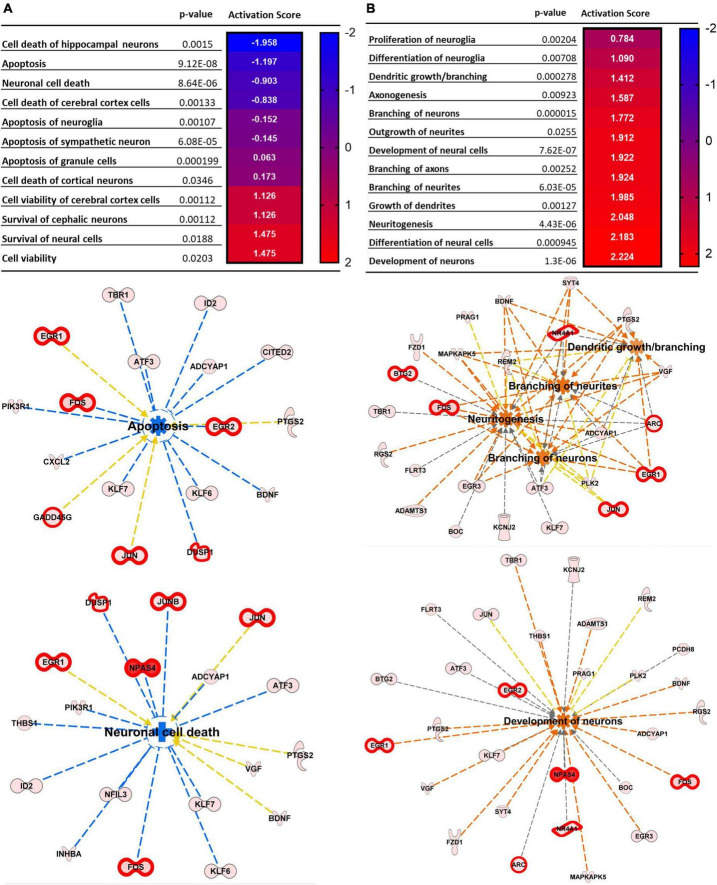
Top Cellular functions generated by IPA analysis of DEGs upregulated in SD animals vs. Contralateral. **(A)** Neural death and survival pathways. **(B)** Cellular proliferation and growth. Top cellular functions generated by IPA analysis of DEGs upregulated in SD animals vs. sham. The heatmaps show the *p*-value and the activation score for each pathway. **(A’,B’)** Graphical representations of the networks and the genes involved in **(A,B)**. The intensity of the color red for each molecule is proportional to the FC of the gene. Orange arrows indicate a positive control of the gene on the related pathway, and the pathway’s name highlighted in orange indicates its activation. Genes with FDR < 0.01 are also highlighted with thicker red outlines. Apoptosis z score –2.003, neuronal cell death z score –1.974, density of spines z score + 1.165, branching of neurons z score + 1.913, dendritic growth/branching z score + 1.090, and development of neurons z score + 2.286.

We next performed the same analyses described above (Figure 1), but comparing tissue exposed to repetitive SDs with the contralateral cortex in the same animals, rather than sham animals. Of the 12,893 mapped genes 149 were significant after unpaired *t*-test followed by Welch’s correction (Figure 3A, black, FC > 1.25, *p*-value < 0.05, *n* = 6, Supplementary Table 1), of these 21 were also significant after FDR correction (FDR adjusted *p*-value < 0.01, FC > 1.5, *n* = 6) (Figure 3A, red). IPA analysis confirmed a high similarity between the sham and contralateral cortex when used as control compared to the SD group. Figures 3B, C show the top scored networks generated from the SD vs. contralateral DEGs list, genes with FDR < 0.01 are also highlighted with thicker red outlines. The top diseases and function are reported in Table 2 and Supplementary Table 2 (compare with Figure 1). Likewise, Figure 4 shows cellular and molecular pathways that are comparable with DEG analysis with sham tissues (compared with Figure 2). These results imply that, at least at the level of gene expression changes after 2 h, the contralateral hemisphere that is not directly invaded by SD can likely serve as an unaffected control.

**TABLE 2:** Predictions of SD-induced changes associated nervous system disease states and functions, in SD ipsilateral vs. contralateral cortex.

Disease and disorders	*p*-value range	# Molecules
Neurological diseases	4.56E-02–2.49E-08	73
Organismal injury and abnormalities	4.56E-02–1.91E-08	73
Psychological disorders	2.85E-02–1.91E-08	35
Hereditary disorders	1.54E-02–1.91E-08	21
Skeletal and muscular disorders	7.75E-03–6.30E-08	20
**Physiological system development and functions**	***p*-value range**	**# Molecules**
Nervous system development and function	4.56E-02–7.04E-07	61
Tissue morphology	4.56E-02–1.07E-09	39
Tissue development	4.56E-02–1.03E-06	49
Organismal development	4.56E-02–5.03E-06	44
Organ morphology	4.56E-02–5.96E-05	16

### 3.4 SD target genes control cell differentiation and proliferation, synaptic plasticity, and stress responses

**FIGURE 5:**
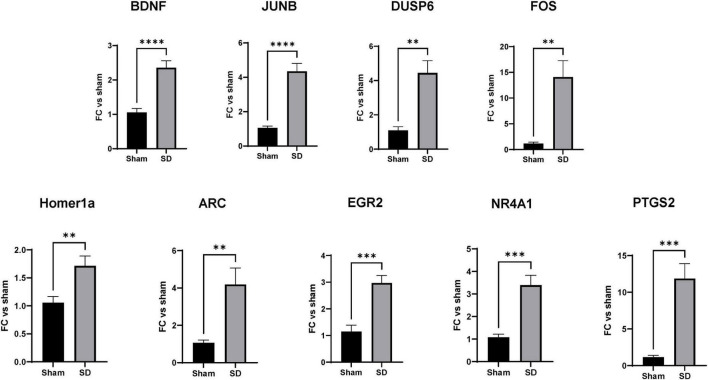
Spreading depolarization (SD) target genes control cell differentiation and proliferation, synaptic plasticity, and stress responses. RT-qPCR validation of SD target genes identified by RNAseq and enrichment analysis. mRNA levels of the target genes were measured as described in section “2 Materials and methods.” Data were analyzed by unpaired *t*-test, followed by Welch’s correction and are represented as mean ± SEM; **p < 0.01, ***p < 0.001, ****p < 0.0001; *n* = 6.

To validate the predicted activation of neuroprotective pathways, we analyzed the expression levels of a subset of SD target genes by qRT-PCR. The genes selected for validation where based on the IPA analysis; indeed, enrichment pathways analysis was used to identify genes functionally related to SD target genes that were not found significant in DE analysis. Based on the IPA results above, we focused on genes regulating 3 cellular functions: (1) cell differentiation and proliferation, (2) synaptic plasticity, and (3) stress responses. qPCR was performed on the same RNA that was subjected to the RNAseq analyses, as well as 3 additional animals for each group. As shown in Figure 5, multiple SDs resulted in significant increases in the expression of genes activating cell proliferation and differentiation such as BNDF, DUPS6, FOS, and JUNB (Figure 5). Considering the predicted activation of neurons and long-term potentiation, as well the increased axonogenesis, growth of neurites, and axon and neurites branching, we then analyzed the expression of genes known for their regulation of synaptic plasticity. As shown in Figure 5 we found significantly increased expression of Homer1a and ARC. Finally, we examined levels of PTGS2, NR4A1, and EGR2 as genes regulating stress and inflammatory responses, showing a significant upregulation.

### 3.5 Target genes showed differential expression depending on the distance from the SD initiation site

**FIGURE 6:**
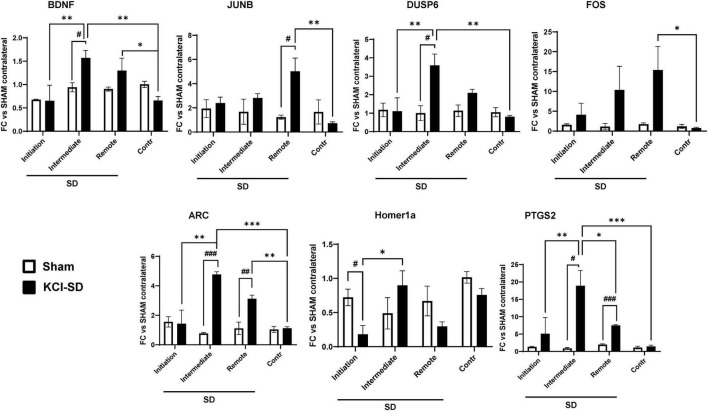
Target genes showed differential expression depending on the distance from the SD initiation site. mRNA levels of the target genes were measured as described in section “2 Materials and methods.” Specifically, we determined the mRNA levels in 4 different regions: (1) SD initiation site, (2) SD intermediate site, (3) SD remote site, and (4) the contralateral hemisphere. Data were analyzed by 2-way ANOVA followed by Tukey’s multiple comparison test, and are represented as mean ± SEM; *p < 0.05, **p < 0.01, ***p < 0.001; and unpaired *t*-test, followed by Welch’s correction and are represented as mean ± SEM; ^#^*p* < 0.05, ^##^*p* < 0.01, ^###^*p* < 0.001; *n* = 3.

Lastly, we tested whether the levels of gene expression varied with distance from the SD initiation site. In this set of studies, KCl was used as the stimulus, as the site of initiation is clearly restricted to small zone of the application of KCl through the burr hole. Thus, 2 h after onset of the initial SD, RNA was extracted from either total contralateral cortex, or from three different regions of the ipsilateral hemisphere: (1) SD initiation site; (2) intermediate site, and (3) remote site (see section “2 Materials and methods” and Supplementary Figure 3). By qRT-PCR we analyzed the levels of the previously identified target genes (see section “3 Results” above) and compared these to the levels in the contralateral hemisphere of SHAM animals. For the majority of the genes examined (BDNF, DUSP6, PTGS2 ARC, and Homer1a), highest expression was detected in the intermediate area (>3 mm from the initiation site; Figure 6) while FOS and JUNB genes showed significantly increased expression in the remote site (>6 mm from the initiation site). Interestingly, when analyzing Homer1a levels, we found a decrease in their expression at the SD initiation site (Figure 6) when compared to the contralateral and intermediate sites (Figure 6). The difference between the contralateral and remote sites for NR4A1 did not reach statistical significance (*p*-value = 0.09) and EGR2 did not show differential expression across regions (Supplementary Figure 3).

## 4 Discussion

This study is the first unbiased analysis of whole transcriptome changes in healthy brain tissue following repetitive spreading depolarization (SD). The main findings are that SD produces a spectrum of gene expression changes that are more diverse than previously thought and which, based on enrichment pathway analysis, likely include multiple molecular networks that promote neuronal survival, recovery and repair. The expression changes were most prominent in areas of SD propagation, implying that it is the depolarization wave itself that produces the changes rather than areas involved in SD initiation. We also show that the contralateral hemisphere, which is not directly involved in the SD, can serve as a good control tissue for DE analyses, even though changes in functional connectivity likely occur during repetitive SD waves. Our findings provide a basis for consideration of a wider and novel range of consequences of SD, in addition to the well-studied acute functional disruption or damage to injured tissues.

Spreading depolarization is a profound activation of brain tissue that leads to depolarization in the order of minutes, and cellular loading of Na^+^, Ca^2+^ (Somjen, 2001; Hartings et al., 2017a), and activation of transcription factors (Ikeda et al., 1994; Sosthenes et al., 2019). In our study we confirmed that expression of immediate early genes is strongly upregulated, including that of established markers of neuronal activity such as FOS, JUNB and ARC (Ikeda et al., 1994; Hermann et al., 1999). SD is a global event that appears to involve all neurons and glia in its path (Somjen, 2001), and thus these markers of cellular activation are expected to be uniformly activated in these cell types after SDs (Lara Aparicio et al., 2022). NMDA receptor and L-type Ca^2+^ channel activation is well established to induce cFOS mRNA and protein expression (Chung, 2015) and the extensive NMDA-dependent and L-type Ca^2+^ channel involvement in SD (Herrera and Robertson, 1990; Shimazawa et al., 1995; Aiba et al., 2012) are likely responsible for cFOS increases observed in the current study. cFOS has been implicated in establishment of synaptic plasticity and memory in rodents (Gallo et al., 2018) and it will be of interest to determine whether block of cFOS signaling might disrupt plasticity that has been observed following SD.

While RNAseq is a powerful tool for identifying genome-wide transcriptional alterations, standard differential gene expression analyses do not themselves leverage known biological gene network relationships. Using enrichment pathway analysis tools, we identified several additional key SD-activated molecules inferred as participants in larger functional networks. Increases in BDNF expression are among the most studied and strongly implicated in ischemic preconditioning and potentially plasticity after SD. BDNF increases were seen here as well. Consistent with previous work (Kokaia et al., 1993; Matsushima et al., 1998; Rangel et al., 2001; Yanamoto et al., 2004), BDNF was one of the genes significantly increased by SD (Figures 5, 6) and was identified in SD-induced pathways related to neuronal differentiation: neuritogenesis, branching of axon sand neurites, and development of neurons (Figures 2, 4). A surprising finding was the extent of predicted pathways involved in neuronal plasticity and regeneration. For example ARC mediates AMPA receptor endocytosis and is required for protein synthesis-dependent LTP and memory formation (Chowdhury et al., 2006). Similarly, Homer1 scaffolding protein controls the insertion of postsynaptic glutamate receptors (Ango et al., 2002; Luo et al., 2012). Homer1a is increased by synaptic activity through the MAP kinase pathway. This type of regulation creates a negative feedback loop in which Homer1a inhibits Homer1b/c ability to scaffold and diminishes synaptic strength by modulation of dendritic spine morphology and AMPA/NMDA receptor activity (Sato et al., 2001; Bottai et al., 2002). This loop has been associated with TBI and epilepsy, particularly, and is thought to block postsynaptic Ca^2+^-mediated excitotoxicity (Cavarsan et al., 2012; Luo et al., 2014). Consistent with these observations, prior work in brain slices and rodent cortex has reported LTP-like synaptic strengthening following SD (Footitt and Newberry, 1998; Berger et al., 2008). In addition previous studies have shown that SD leads to synaptic strengthening (Footitt and Newberry, 1998; Berger et al., 2008; Theriot et al., 2012) as well as adult neurogenesis (Urbach et al., 2017) and preconditioning (Matsushima et al., 1998; Yanamoto et al., 2004; Shen et al., 2016). It will be of interest to determine whether the time course of associated gene expression changes correspond to variations in physiological recordings and how the expression of these genes change under different metabolic conditions surrounding ischemic infarcts.

Consistent with previous reports, either from gene array studies or targeted expression analysis (Urbach et al., 2006; Sintas et al., 2017) markers of inflammation were upregulated. The functional consequences of inflammatory responses to SD are likely to be wide-ranging and include modification of pathological outcomes in stroke, headache and also susceptibility to additional SD events (Chen and Ayata, 2017; Varga et al., 2020). Our results are consistent with prior reports of robust increases of the pro-inflammatory mediator COX2 (Kaido et al., 2012; Takizawa et al., 2020; Volobueva et al., 2023). COX-2 inhibition prevented SD-induced increases in non-REM sleep (Cui et al., 2008) and it will be of interest to determine whether selective COX-2 inhibition modifies other consequences of SD in healthy and vulnerable brain tissues. We did not see significant increases in some other inflammatory mediators that had been previously reported (e.g., TNF-α, IL-1β, IL6), perhaps because the time course for peak increases in these mediators is later than the 2-h time point used in the present study (Takizawa et al., 2020). This time point was chosen based on (1) prior expression analysis confirming peak increases at 2 h for most genes of interest (Kokaia et al., 1993; Karikó et al., 1998; Rangel et al., 2001) and (2) clinical recordings establishing clusters of SDs (usually defined as three or more events within 2 h) as being clinically significant (Dreier et al., 2006; Hartings et al., 2020). Prior work (Takizawa et al., 2020) has shown that some inflammatory mediators can be detected following a single SD (measured at 1 h), while others peak at 24 h (Matsushima et al., 1998; Jander et al., 2001; Volobueva et al., 2023). It remains to be determined which of the genes seen here may peak earlier or at later times points. In addition, single-cell sequencing and gene expression analysis in different cell types could be a useful future direction to determine the specific involvement of microglia, astrocytes and other cell types in these and other responses to SD, especially in injured tissues.

A second surprising finding came from pathway analysis of genes associated with neuronal injury or apoptotic cell death. The pathways analysis indicated a *downregulation* of these pathways, based on differential expression of molecular components of cell viability or apoptotic pathways such as: BDNF, FOS, EGR1 and 2, ATF3, NAPS4, CXCL2, and MAPKs (Figures 2, 4). This raises the possibility that the extreme metabolic challenge of SD may be capable of promoting cell survival by decreasing cell death pathways. This does not mean that SDs are not causing neuronal injury—it means instead that SD is likely able to kill neurons due to extreme metabolic depletion, but in neurons that are able to recover from that acute insult, gene expression changes favor survival, including downregulation of cell death pathways. Interestingly, recent work has suggested that SD may not directly contribute to injury or perhaps contribute to benefit in some experimental conditions (Fischer et al., 2023; Sugimoto et al., 2023). While further studies will be required to test if SDs is sufficient for a positive outcome after stroke or brain injury; this work shows that in tissues with sufficient perfusion, SD itself can induce the expression of neuroprotective pathways. An important future direction will be to determine whether DEGs seen here following SDs in healthy tissues are also observed in injured brain, and whether or not putative pathways involved in recovery and repair are more or less strongly expressed in tissue at risk for ischemic or traumatic injury.

Another important experimental consideration that is somewhat unique for SD studies, is the choice of control tissue. SDs can propagate throughout a hemisphere but generally do not cross the midline and invade the contralateral hemisphere. For this reason, the contralateral hemisphere is often used as a control tissue for DEG studies (Shen and Gundlach, 1998; Wiggins et al., 2003; Volobueva et al., 2023), with the assumption that it is unaffected by SD. However, a well-established consequence of SD is a spreading depression of synaptic transmission throughout the affected cortex and, because of the connectivity between hemispheres (Leao, 1944; Vinogradova et al., 2021), an abrupt silencing of such large territory is expected to have consequences on circuit activity throughout the brain. We therefore tested whether the results of DEG analysis were different if the contralateral hemisphere was used instead of different animals in which no SD was generated (shams, as used in Figures 3, 4). Interestingly, when tested at this 2 h time point, there was a very similar (although not identical) set of DEGs in both control tissues, implying differences in connectivity only cause minor differences in expression in the contralateral hemisphere compared with the changes produced by propagation of SD through the ipsilateral cortex. This suggests that the benefits of using control tissue from the same animal (to reduce inter-animal variability) may outweigh small changes in state for most SD studies.

Gene expression studies of SD have specific experimental challenges, related to the unusually extreme global disruptions produced by the events. Firstly, experimentally induced SDs require coordinated depolarization of a volume of brain tissue that can be accomplished by a range of methods. Two commonly used methods (focal application of KCl and optogenetic stimulation) of SD were used here and combined, to increase generalizability of results. We then tested whether a microdissection and qPCR would show differences in propagation areas, as compared with the initiation sites. Interestingly, the largest expression changes were observed in the propagation regions (Figure 6), despite the fact that depolarization may be most intense at the initiation site. Factors released by SD or intracellular pathways activated by the combination of factors released at the wave front may be responsible. It also suggests that any injury or other damage produced at an initiation site was not the driver of changes observed in the study. Spatial genomics approaches (Ibarra-Lopez et al., 2022) will be useful to further investigate the distribution of changes of larger gene sets with distance from SD initiation, and with respect to differential vascular perfusion, for example.

Our study has several limitations. Firstly, since general anesthesia may impact spreading depolarization frequency, propagation speed and sensitivity to pharmacological suppression (Kudo et al., 2008) it is possible that DEG changes may be influenced by the choice of anesthetic. While no data on urethane has been published at the time, prior literature shows gene expression changes in immediate-early genes induced by Isoflurane and other anesthetics (Bunting et al., 2016; Upton et al., 2020; Shpetko et al., 2023). Future studies utilizing awake freely moving animals could possibly improve the translatability of pre-clinical findings, including using novel approaches currently under development (Masvidal-Codina et al., 2021). Our study also utilized female mice, in order to improve reproducibility with optogenetic SD stimulation (Chung et al., 2019). Possible sex-differences in DEGs following SD were not addressed. Finally, as discussed above, we chose a single time point for DEG analysis (2 h following SD clusters). Additional changes may be observed at earlier or later time points, including changes that may contribute to circuit modification or adaptation that occur over longer time frames. In this context, we found that the contralateral hemisphere could serve as an appropriate control for SD-induced DEG changes observed at 2 h in the present study, but it is possible that functional connectivity differences could lead to larger DEG changes at other time points, particularly in awake, freely moving animals.

In conclusion, these findings provide a basis for considering long-term changes due to SD that can influence outcomes in a range of neurological disorders. While SDs can clearly be damaging to vulnerable brain tissue, our work shows the changes in gene expression may also promote neuronal survival in some areas. If the predicted pathways involved in neuronal differentiation and growth are manifest in surviving peri-infarct tissues, for example, then there may be benefit to preserving these effects while preventing deleterious consequences of SD. These results also emphasize the importance of experimental design for SD studies. Finally, SDs that are experimentally generated outside a zone of vulnerable tissue may have the ability to activate pro-survival pathways predicted in the current study and thereby contribute to apparent paradoxical results in experimental models of stroke. Further studies will be essential to determine whether SD induction of neuronal survival pathways are sufficient to improve recovery and outcomes in stroke and brain injury.

## Data availability statement

The datasets presented in this study can be found in online repositories. The names of the repository/repositories and accession number(s) can be found in the article/Supplementary material.

## Ethics statement

The animal study was approved by the University of New Mexico Health Sciences Center Institutional Animal Care and Use Committee. The study was conducted in accordance with the local legislation and institutional requirements.

## Author contributions

MD: Conceptualization, Data curation, Investigation, Methodology, Writing – original draft. JW: Conceptualization, Writing – review and editing. NP-B: Writing – review and editing. AC: Writing – review and editing. RM: Writing – review and editing, Methodology. DL: Writing – review and editing. CS: Funding acquisition, Resources, Supervision, Writing – review and editing, Conceptualization.

## Footnotes

1
https://imagej.nih.gov/

2
https://pga.mgh.harvard.edu/primerbank/

3
https://www.qiagenbioinformatics.com/products/ingenuity-pathway-analysis
