# Supplementary material for: Repetitive spreading depolarization induces gene expression changes related to synaptic plasticity and neuroprotective pathways
Source: Front Cell Neurosci. 2023 Dec 14;17:1292661. doi: 10.3389/fncel.2023.1292661 (PMC10757627; doi:10.3389/fncel.2023.1292661)
Supplement: Supplementary file 5 [file Table_5.DOCX]

Supplementary Material

Repetitive Spreading Depolarization induces gene expression changes related to synaptic plasticity and neuroprotective pathways.

Michela Dell’Orco ^1^, Jordan E. Weisend ^1^, Nora I. Perrone-Bizzozero ^1^, Andrew P. Carlson ^2^, Russell A. Morton ^1^, David N Linsenbardt ^1^ & C. William Shuttleworth ^1^

*** Correspondence:** C. William Shuttleworth, Ph.D.[bshuttleworth@salud.unm.edu](mailto:bshuttleworth@salud.unm.edu)

# Supplementary Data

# Supplementary Figures

## Supplementary Figure 1 Schematic representation of the SDs induction: A) SD induction was with either focal application of KCl (NaCl for sham controls, n=6) in C57Bl/6 mice or with optogenetic stimulation (2 mW for 20 seconds) in Thy1-ChR2-YFP mice (n=6). SDs were induced repetitively (4 SDs in 2 hours at 30 min intervals) in healthy, female mice and SDs were confirmed with Intrinsic Optical Signal imaging B) iOS images of the SDs propagating through the left hemisphere; after KCl application, or Optogenetic stimulation

## Supplementary Figure 2 Differential Expression (DE) analysis. We performed unpaired t-test followed by Welch’s correction; significant genes (FC > 1.25, p value <0.05, n=6) are represented in black. Consequently, we performed DESeq2 analysis (Ge, Son, and Yao 2018); no genes significant after False Rate Discovery correction (FDR <0.01, n=6).

## Supplementary Figure 3 A) Representation of the localization of the micro dissected tissues collected for qRT-PCR B) GAPDH levels in RNA sequencing samples are expressed as FPKM. No differences in GAPDH were found after either KCl- or optogenetic- induced SDs. Thus, GAPDH represents a valid hkg for qRT-PCR validation. Data were analyzed by unpaired t-test, followed by Welch’s correction and represented as mean ± SEM, n=3. C) Target genes showed differential expression depending on the distance from the SD initiation site. mRNA levels of the target genes were measured as described in Material and Methods. Specifically, we determined the mRNA levels in 4 different regions: 1) SD initiation site, 2) SD intermediate site, 3) SD remote site, and 4) the contralateral hemisphere. Data were analyzed by 2-way ANOVA followed by Tukey’s Multiple Comparison test and are represented as mean ± SEM; n=3.

# Supplementary Tables

## Supplementary Table 1 DEGs

## Supplementary Table 2 Diseases and Functions

## Supplementary Table 3 Netwoks

## Supplementary Table 4 cellular functions

1. **Videos**

## Video 1 KCL SD

## Video 2 Optogenetics SD

**
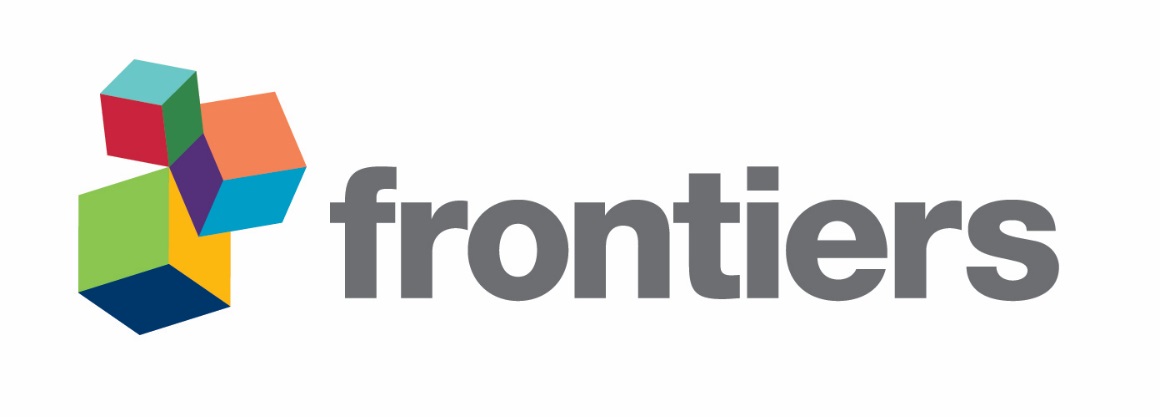
**
