# Supplementary figures and images for: Repetitive spreading depolarization induces gene expression changes related to synaptic plasticity and neuroprotective pathways
Source: Front Cell Neurosci. 2023 Dec 14;17:1292661. doi: 10.3389/fncel.2023.1292661 (PMC10757627; doi:10.3389/fncel.2023.1292661)

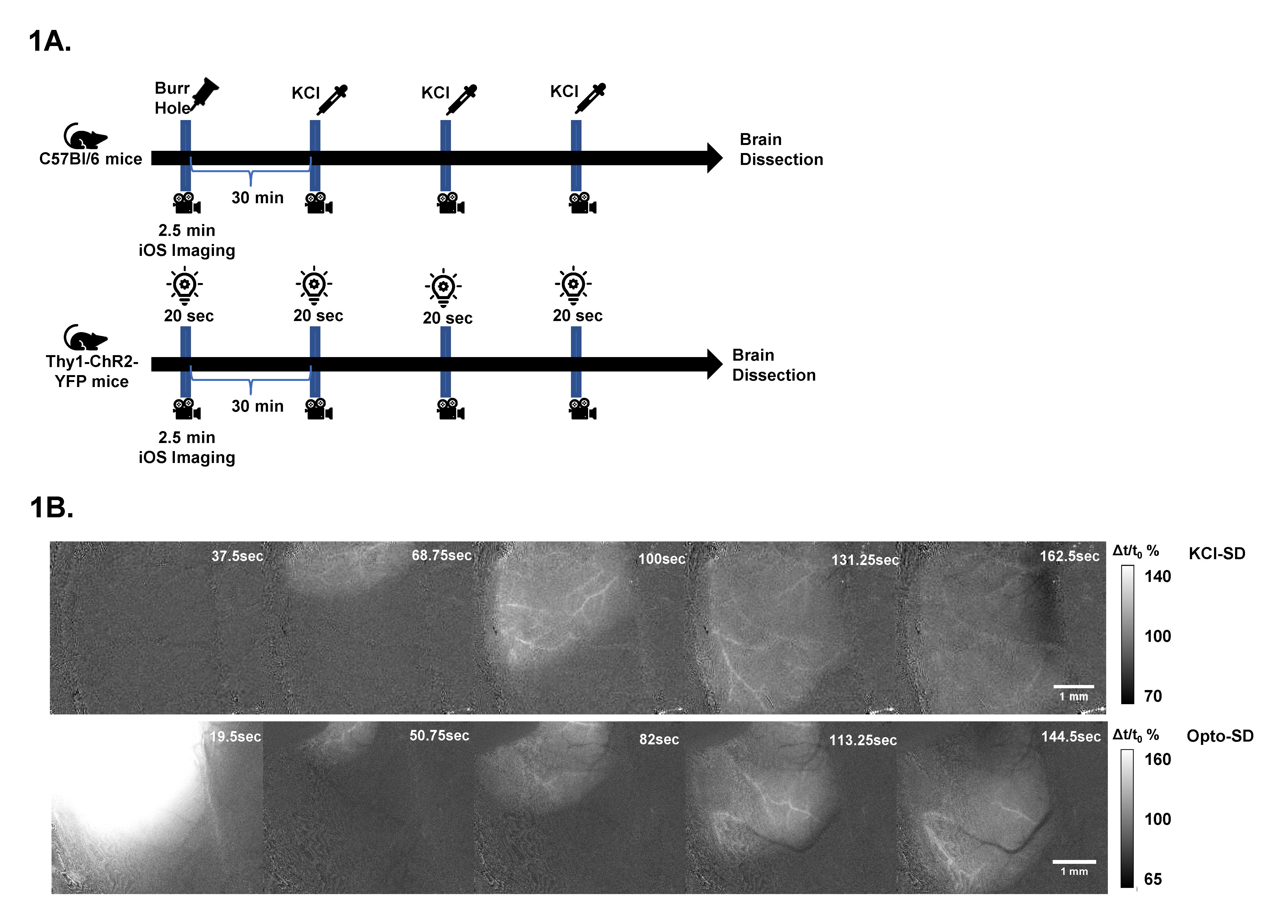

Supplement: Supplementary file 8 [file Image_1.TIF]

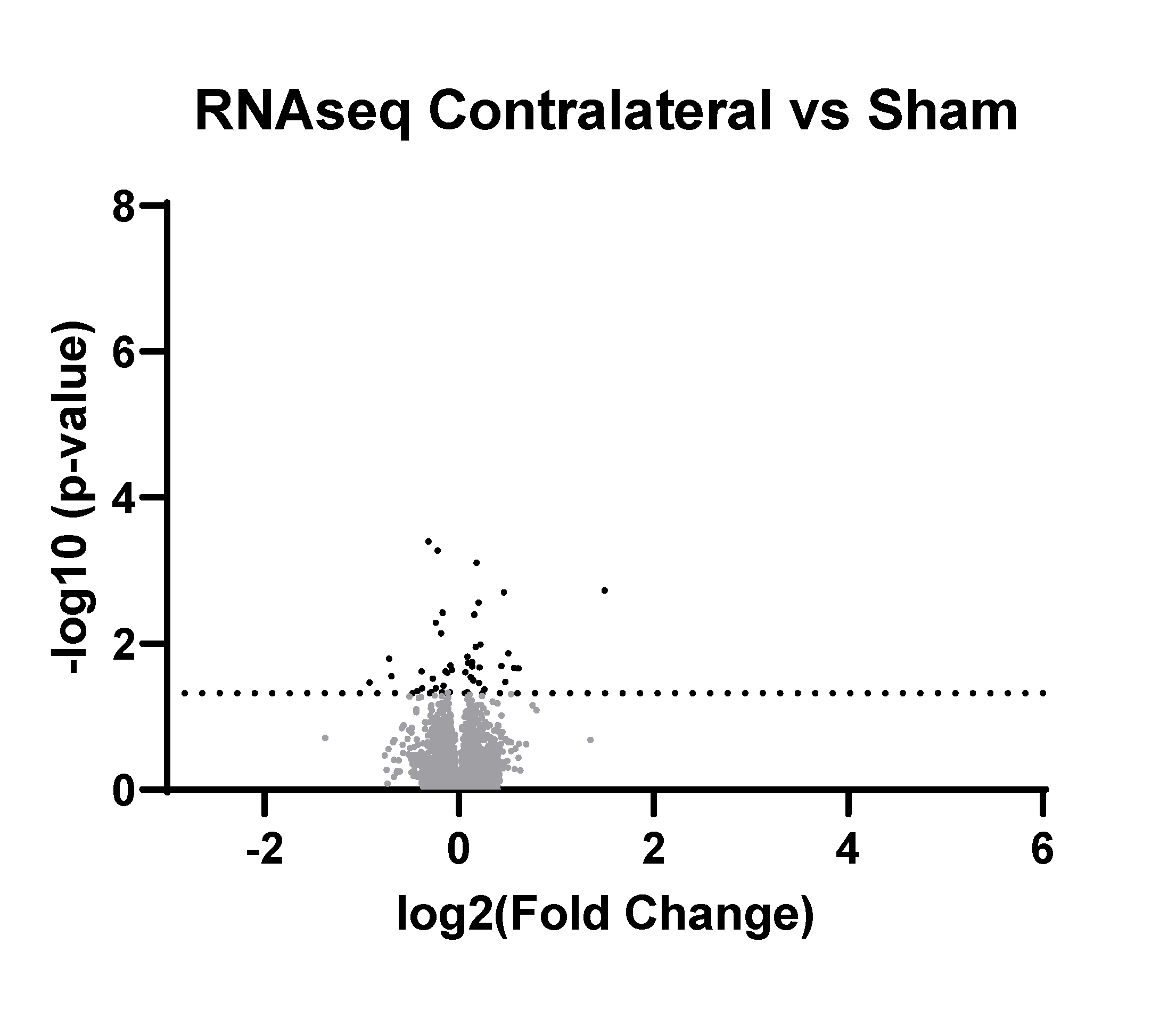

Supplement: Supplementary file 9 [file Image_2.TIF]

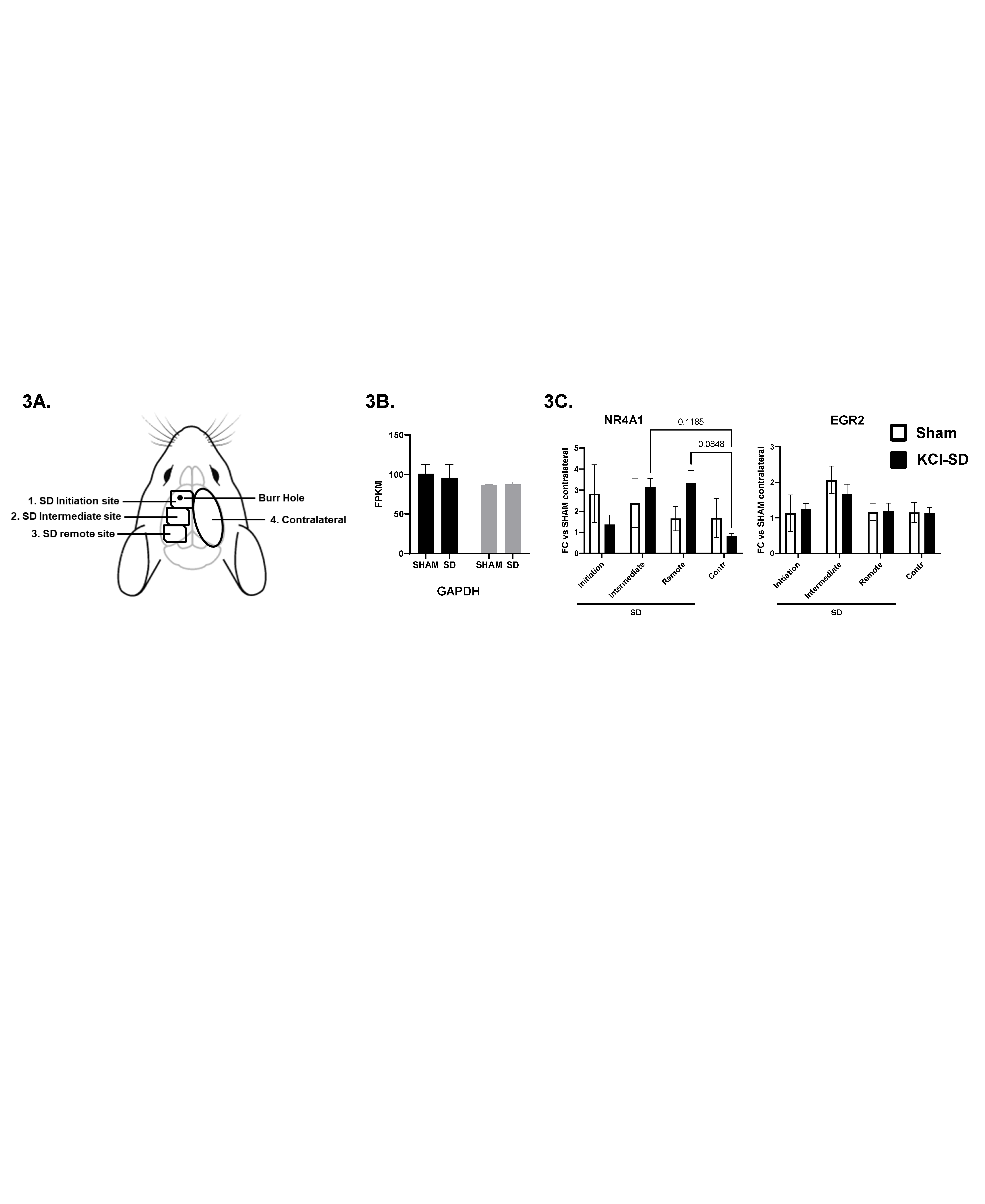

Supplement: Supplementary file 10 [file Image_3.TIF]
